# ﻿Immune priming in *Armadillidiumvulgare* against *Salmonellaenterica*: direct or indirect costs on life history traits?

**DOI:** 10.3897/zookeys.1101.77216

**Published:** 2022-05-18

**Authors:** Cybèle Prigot-Maurice, Charlotte Depeux, Hélène Paulhac, Christine Braquart-Varnier, Sophie Beltran-Bech

**Affiliations:** 1 Université de Poitiers, Laboratoire Écologie et Biologie des Interactions, UMR CNRS 7267, 3 rue Jacques Fort, TSA 51106, F-86073 POITIERS Cedex 9, France Université de Poitiers Poitiers France

**Keywords:** Crustacea, haemocytes, immune priming, isopod, reproduction, senescence, survival, trade-off

## Abstract

Invertebrate immune priming is defined as an enhanced protection against secondary pathogenic infections when individuals have been previously exposed to the same or a different pathogen. Immune priming can be energetically costly for individuals, thus impacting trade-offs between life-history traits, like reproduction, growth, and lifetime. Here, the reproductive cost(s) and senescence patterns of immune priming against *S.enterica* in the common woodlouse *A.vulgare* (Crustacea, Isopoda) were investigated. Four different groups of females were used that either (1) have never been injected (control), (2) were injected twice with *S.enterica* (7 days between infections), (3) were firstly injected with LB-broth, then with *S.enterica*, and (4) females injected only once with *S.enterica*. All females were allowed to breed with one non-infected male and were observed for eight months. Then, the number of clutches produced, the time taken to produce the clutch(es), the number of offspring in each clutch, the senescence biomarkers of females, and parameters of their haemocytes were compared. The result was that immune priming did not significantly impact reproductive abilities, senescence patterns, and haemocyte parameters of female *A.vulgare*, but had an indirect effect through body weight. The lighter immune primed females took less time to produce the first clutch, which contained less offspring, but they were more likely to produce a second clutch. The opposite effects were observed in the heavier immune primed females. By highlighting that immune priming was not as costly as expected in *A.vulgare*, these results provide new insights into the adaptive nature of this immune process.

## ﻿Introduction

Because fighting pathogens is a real challenge for all living organisms, they have developed an important and complex biological process, the immune system (Siva-Jothy et al. 2005; Danilova 2006), which is commonly divided into the innate immunity, which is found in all organisms and is based on non-specific recognition of intruders (Medzhitov and Janeway 2000), and the adaptive immunity, which is observed only in jawed vertebrates and allows the remembering of previous encounters with pathogens to prepare the immune response for a second exposure. Adaptive immunity mainly rely on specialised cells, the lymphocytes (Cooper and Alder 2006). Because invertebrates only have the innate immunity, it has long been thought that they were not able to express memory-like features following subsequent infections with pathogens (Janeway and Medzhitov 2002; Cooper and Eleftherianos 2017). Recent findings reported the other way around: in more than 40 invertebrate species, individuals improve their survival abilities upon a second pathogenic infection when they have been previously exposed to the same, or another, pathogen (Little and Kraaijeveld 2004; Kurtz 2005; Milutinović and Kurtz 2016; Netea et al. 2019). In invertebrates, this immune process is called “immune priming”. Immune priming can last for a few days to several months depending on the species (Milutinović and Kurtz 2016) and is mediated by three, non-exclusive, major mechanisms: (1) the recalled immune response, involving a first immune response that returns to basal levels before the second exposure to pathogen, then a second enhanced immune response with the same immune activity (Contreras-Garduño et al. 2016); (2) the immune shift, that firstly involves one type of immune activity (e.g., cellular encapsulation), then a different kind of immune activity during the second infection (e.g., humoral expression of antimicrobial-related genes; Pinaud et al. 2016); and (3) the sustained immune response, which lasts from the first to the second infection event (reviewed in Coustau et al. 2016; Melillo et al. 2018).

Although immune priming is advantageous in terms of survival ability, its expression could be costly for individuals, particularly when it relies on the long-lasting sustained immune response (Moret 2003; Contreras-Garduño et al. 2014, 2019; Coustau et al. 2016; Shikano et al. 2016; Khan et al. 2019). According to the theory of life-history traits, organisms must allocate their limited energy to different biological functions by making trade-offs. Individuals which invest a lot of energy in reproduction during their early life may have lower survival abilities (Descamps et al. 2006; Lemaître et al. 2015). On the opposite side, individuals investing in their somatic maintenance (including pathogen clearance) may have less energy for other biological functions, decreasing thus their reproductive ability (Rolff and Siva-Jothy 2003; Schmid-Hempel 2005; Luu and Tate 2017). In *Anophelesgambiae* (Giles 1902), immune primed females which clear the second infection of *Plasmodiumberghei* (Vincke & Lips, 1948) do not produce eggs, and those which do not successfully eliminate *P.berghei* exhibit a lower hatching rate compared to non-primed females (Contreras-Garduño et al. 2014). In addition, the immune responses of invertebrate individuals sometimes involve cytotoxic compounds (e.g., phenoloxidase products, reactive oxygens species), which can damage the cells and tissues of the host, especially if the immune response is systemic (Moret 2003; Sadd and Siva-Jothy 2006). However, the investment in immune functions can sometimes enhance the reproductive abilities of individuals, by the terminal investment strategy (Adamo 1999; Duffield et al. 2017; Luu and Tate 2017). The terminal investment strategy refers to the direct allocation of the remaining energy in reproduction when the individual’s probability of dying is expected to increase (Creighton et al. 2009; Duffield et al. 2017). This strategy can also be triggered by the senescence patterns of individuals because natural ageing or advanced senescence caused by environmental/physiological stress results in somatic deterioration and reduced lifetime, like immune response can do (Comfort 1964).

Nevertheless, several studies did not observe the impact of repeated infections with pathogens on the reproductive abilities of individuals, within the same or the subsequent generations (Faria et al. 2015; Gupta et al. 2016; Prakash et al. 2022). In a recent experimental evolution study, *Triboliumcastaneum* (Herbst, 1797) lineages that evolved with two consecutive infections with inactivated and living *Bacillusthuringiensis* (Berliner, 1915) for 14 generations displayed lower reproductive costs compared to lineages that evolved only with inactivated *B.thuringiensis* (Prakash et al. 2022). Females of *Tenebriomolitor* (Linnaeus, 1758) previously challenged with *Arthrobacterglobiformis* (Conn & Dimmick, 1947) or *B.thuringiensis* produce as many eggs as the non-primed females, and these eggs have higher hatching rates (Dhinaut et al. 2018). These experiments highlight that immediate reproduction is not always influenced by the increased immune protection across generations. Most empirical studies that investigated the impact of immune priming on the reproduction of females analysed their reproductive abilities after the first infection and on the next generation. Although these results provide important knowledge on the evolutionary trade-offs, with reproduction resulting from the first immune stimulation, we are lacking knowledge about the reproductive cost(s) of females that survived the first immune stimulation and the second, lethal pathogenic infection with living pathogens. We also lack information on whether immune priming and the following reproductive event(s) affect the senescence pattern of individuals, although they should indicate the adaptive features of such immune process.

Among the numerous species in which immune priming has been observed, the common woodlouse *Armadillidiumvulgare* (Oniscidea, Isopoda, Crustacea) is an appropriate model to investigate this issue. *Armadillidiumvulgare* (Latreille, 1804) mount an immune priming response with two subsequent infections of living *Salmonellaenterica* (Theobald Smith, 1855) injected seven days apart (Prigot-Maurice et al. 2019, 2021). The underlying mechanism is expected to be a sustained immune response of primed individuals, which display long-lasting, higher viability of haemocytes compared to non-primed individuals (Prigot-Maurice et al. 2019). This assumption is supported by the persistence of *S.enterica* in the haemolymph of the primed individuals between the infections. Although primed individuals would die faster than those receiving only one injection, this study shows that they are even more able to survive and deal with very high titres of pathogenic bacteria (Prigot-Maurice et al. 2019). Hence, the energetic costs of mounting immune priming and simultaneously repairing the tissues damaged by the persistent infection of *S.enterica* would be very high in this host-pathogen system. The reproductive events in female of *A.vulgare* also require important energetic resources (Warburg et al. 2001). To reproduce, females develop exoskeletal extrusions which form a ventral brood pouch, named marsupium. Females oviposit and incubate their eggs in the marsupium throughout embryogenesis for ca. a month (Surbida and Wright 2001). On average, females produce one or two marsupia (i.e., one or two clutches) per year, during the reproductive season (Lawlor 1976; Dangerfield and Telford 1995). This extensive maternal care partly determines the fecundity of females, because larger females produce larger marsupium in which they can incubate more eggs than smaller females (Sutton et al. 1983; Antoł and Czarnoleski 2018; Durand et al. 2018). *Armadillidiumvulgare* exhibits indeterminate growth, allocating energy in growth throughout its lifetime. In this species, the size of individuals is closely correlated to their weight, and the largest and heaviest individuals are usually the oldest (Depeux et al. 2020b). In natural populations, the average lifespan of *A.vulgare* is two years (Paris and Pitelka 1962). Females in the field reproduce until three years old (Dangerfield and Hassall 1992), although most individuals die at approximately the age of one year old, following the first reproductive season (Paris and Pitelka 1962). In addition to these biological features, *A.vulgare* live in an environment rich in microbial density and diversity (Warburg et al. 1984; Broly et al. 2013; Ranjard and Richaume 2001; Zimmer 2002), where the risk of exposure to pathogens related to its lifespan is expected to be high (Little and Kraaijeveled 2004). Hence, the use of *A.vulgare* opens up the possibility to easily observe evolutionary trade-offs between somatic maintenance and reproductive investment, with the main objective being the exploration of adaptive features of immune priming. Recently, several biomarkers have been identified to estimate the cellular senescence patterns in *A.vulgare* (Depeux et al. 2020b). These biomarkers allow us to determine the senescence patterns of individuals of the same age. They may thus provide important clues about the cost(s) of maintaining survival and/or reproductive ability under stressful conditions, like infections with pathogens.

In this study, we explored the impact of immune priming with *S.enterica* on the reproductive ability and the resulting senescence patterns of *A.vulgare*. Our objectives were: (1) to test whether mounting an immune priming response affects the reproduction of females that successfully survived two consecutive infections with living *S.enterica*, and (2) to explore to what extent immune priming and reproduction change the senescence patterns of individuals, by using two senescence biomarkers: the β-galactosidase activity and the size of the viable haemocytes (described in Depeux et al. 2020b). Because reproduction is known to negatively affect the immune system (Lawniczak et al. 2007), we also (3) compare the total concentration and viability of haemocytes after the last reproductive event of females. To do so, we used females firstly injected (i.e., primed) either with a low living dose of *S.enterica*, sterile LB-broth, or non-primed females. Seven days later, we injected all these females with LD_50_ of *S.enterica*. Since the fecundity of terrestrial isopod females is correlated with their size (Sutton et al. 1983; Antoł and Czarnoleski 2018; Durand et al. 2018) and immune responses may decrease the growth and/or body weight of the females (Moret 2006; Bascuñán-García et al. 2010; Kelly 2011), we weighed the females that survived the sublethal infection of *S.enterica* and allowed them to mate with one virgin, non-injected male. We also added females that have never been injected with *S.enterica* (control group) but maintained under the same experimental conditions as the females in the other treatments. To compare the reproductive cost of surviving females that mounted immune priming or not, we measured the probability of producing one or two clutches, the time taken to produce these clutches, and the number of viable offspring in each clutch. After the reproductive event(s), we analysed the β-galactosidase activity and the haemocyte parameters (size of viable haemocytes, concentration, and viability of haemocytes) of all females.

## ﻿Materials and methods

### ﻿Biological model and bacterial cultures

In this experiment, we used the same *Armadillidiumvulgare* line used in the study of Prigot-Maurice et al. (2019). The virgin females with an age of one year (± 2 months) came from laboratory cross-breeding of individuals initially sampled at Helsingør, Denmark (1982). During the breeding period, females were kept in moistened potting mix supplied with linden leaves and carrot slices ad libitum in 10 × 30 cm boxes, under natural photoperiod and room temperature.

To perform the infections, we used the *Salmonellaenterica* serovar *typhimurium* J18 strain (Verdon et al. 2016). The cultures of *S.enterica* were performed as described in Braquart-Varnier et al. (2015). Briefly, the *S.enterica* strain came from one frozen glycerol stock, streaked on Luria-Bertani Broth plates (25 g.L^-1^ of LB base, Invitrogen 12795-027 supplemented with 15 g.L^-1^ of agar-agar, Fisher BioReagents, BP1423-2) at 37 °C overnight. One Colony Forming Unit (CFU) was then added to 5 mL of liquid LB broth at 37 °C, 180 rpm overnight. 100 µL of this *S.enterica* culture were grown in 3 mL of fresh LB broth under the same conditions to reach an optical density of 0.8 (600 nm). 1 mL was centrifugated (2 min, 4 °C, 13,000 g) and the bacterial pellet resuspended in 100 µL of LB broth. This first tube contained 10^6^*S.enterica* for 100 nL of injection (LD_50_ dosage). Serial dilutions were then achieved to obtain the dosage for the first injection (10^3^*S.enterica* for 100 nL). To control the quantity of injected *S.enterica*, we diluted four times more the first tube, reaching the concentration of 1 bacterium per µL. We plated 100 µL of this solution onto LB agar plate and we counted the number of CFU after an overnight culture at 37 °C.

### ﻿Experimental design

**Figure 1.**
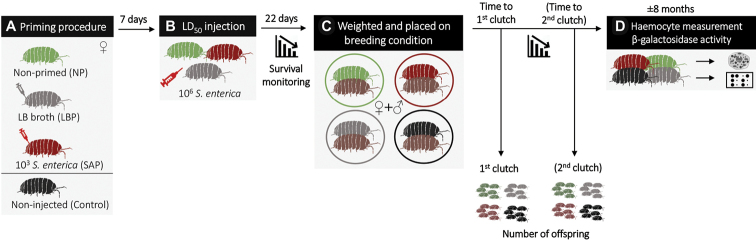
The experimental procedure **A** the priming procedure was to inject females either with a low dose of living *S.enterica* (SAP, in red) or sterile LB Broth (LBP, in grey). The non-primed (NP, in green) females did not receive the first injection **B** SAP, LBP, and NP females received the second, LD50 injection of living *S.enterica*. Control females (in black) were never injected **C** all females (SAP, LBP, NP, control) were allowed to reproduce individually in a box with one virgin, non-injected male (brown woodlouse). We checked the survival rates of females, the number of clutches (1 or 2), the time to produce each clutch (number of days), and the number of offspring in each clutch **D** regularly, we sampled and dissected females that produced the second clutch to analyse haemocytes and β-galactosidase activity. Brackets indicate that not all females produced a second clutch. Approximately eight months later, we waited for the last females to produce their second clutch, and then sampled and dissected the remaining females that produced only one clutch.

Firstly, we performed the priming procedure on three females’ treatments: either primed (i.e., primo-injected) with the low dose of living *S.enterica* (SAP, for *S.enterica*-primed), with sterile LB broth (LPB, for LB-primed) or without priming injection (NP, for non-primed; Fig. 1). We added a fourth treatment in which the females have never been injected (control females). We used a total of 123 females, including 32 SAP, 33 LBP, 33 NP, and 25 control. Seven days after the priming procedure, SAP, LBP and NP females were all injected with a LD_50_ of *S.enterica*, and their survival rates were monitored for 22 days. Surviving females (SAP = 26, LBP = 27, NP = 20, control = 25) were weighted and placed onto a box (5×8cm) with one virgin non-injected male (Fig. 1). Each pair of individuals were kept on moistened potting mix with linden leaves and carrot slices ad libitum under a stimulating photoperiod (18:6 D/N) at 21 °C.

Every three days for ca. eight months, we measured the survival rate and the physiological states of all females by observing their ventral faces. The females that were about to lay eggs developed a marsupium following a parturial moult, which is observable under a binocular loupe (Moreau and Rigaud 2002). Once females were ready to deliver their offspring, they were placed alone in a box on moist paper. For each female, we counted the number of clutches (one or two), the number of days that they took to produce each clutch (i.e., from the contact with the male to the delivery of offspring; or the time between the first and the second clutch), the number of offspring produced in each clutch and the total number of produced offspring (Fig. 1). After the second clutch (when it occurred), we washed the females (0.28% NaClO then water) and measured the viable haemocyte size, viability (% of living cells), and concentration. We dissected the nerve cord to quantify the β-galactosidase activity. Because not all females produced a second clutch, we decided to sample and dissect the females that had only one clutch at the same time we did this for the last females which produced the second clutch (ca. eight months after the beginning of the experiment; Fig. 1).

### ﻿Priming procedure and LD_50_ injection

The priming procedure with *S.enterica* was performed as described in Prigot-Maurice et al. (2019). Briefly, females were washed (0.28% NaClO then water) and injected dorsally between the 6^th^ and 7^th^ pereon segment under sterile conditions, using a Drummond TM Nanoject (3–000-205A). Injections were performed by two successive injections of 50 nL, either with 10^3^ ± 1.10^2^ living *S.enterica*, or sterile LB Broth. All females (SAP, LBP, NP, and control) were individually isolated in a plastic box, on moist paper without food. We controlled the injected dosage as described above. Seven days later, the SAP, LBP, and NP females were injected with 10^6^ ± 1.10^5^ living *S.enterica* in 100 nL of LB broth (i.e., LD_50_, dosage to kill 50% of individual in seven days) following the same method as for the priming procedure. Females were replaced on their box, and we monitored their survival rates (i.e., immune priming protection) every eight hours for seven days. We adjusted the humidity of each box daily. Then, females were placed alone on moistened potting mix with linden leaves and carrot slices ad libitum for fifteen days and we added the virgin, non-injected male.

### ﻿Haemocyte analysis

After their second clutch, females were washed (0.28% NaClO then water). The total haemocyte concentration (number of haemocytes per µL of haemolymph, regardless of the haemocytes’ type), the viability of haemocytes (% of living haemocytes), and the size of viable haemocytes (µM) were measured as described in Sicard et al. (2010). Three µL of haemolymph were sampled by piercing the females in the 6^th^ tergite with a sterile needle and diluted in 15 µL of MAS solution (27 mM sodium citrate, 336 mM NaCl, 115 mM glucose, 9 mM EDTA, pH 7; Herbinière 2005). We added 6 µL of Trypan blue (0.4%) and 10 µL of the resulting sample were deposited in the counting chamber of the automated cell counter Countess^TM^ Version B (Invitrogen). We waited for the last females to produce their second clutch (ca. eight months) to sample and dissect females which produced only one clutch.

### ﻿β-galactosidase activity

After collecting their haemolymph, all females used for haemocyte analysis were dissected in Ringer solution (135 mM sodium chloride, 2 mM potassium chloride, 2 mM calcium chloride, 2 mM sodium bicarbonate) to collect their nerve cords. The β-galactosidase activity was measured as described in Depeux et al. (2020b). Briefly, each nerve cord was deposited in 300 µL of Lyse buffer 1X (5 mM Chaps detergent, 40 mM citric acid, 40 mM sodium phosphate, 0.5 mM benzamidine, and 0.25 mM PMSF, pH 6), ground manually, and centrifugated for 30 min, 15 000 g, 4 °C. The supernatant was collected and proteins were assayed using the Bicinchoninic Acid Assay (Thermo Fisher Scientific) and standardised at 0.04 mg.mL^-1^ to perform the β-galactosidase activity assay on the same titres of proteins. Subsequently, 100 µL of each protein sample was added to the MUG reagent solution (4-methylumbelliferyl-D-galactopyranoside) in a 96-well microplate. The fluorescence produced by the synthesis of 4-methylumbelliferone (4-MU) was measured by the multimode Mithrax microplate reader (LB940 HTS III, excitation filter: 120 nm, emission filter: 460 nm; Berthold Technologies) for 120 minutes. Two technical replicates were set up for each sample to obtain the result by averaging the replicates’ values.

### ﻿Statistical analysis

All statistical analysis were performed with RSTUDIO (v.1.4; R Core Team 2017). We compared the survival rates of females after the LD_50_ and during the reproductive period by using two global mixed effects Cox proportional hazard regression models, built with coxme package (Therneau et al. 2003). We entered the females’ treatment (SAP, LBP, NP, control) as fixed effect, and Hazard Ratios (HR) were estimated thanks to the instantaneous risks of death between NP or control and other females’ treatments (SAP, LBP, and NP).

Body weight differences of females before reproduction were tested with a linear mixed effects model built with lme4 and *car* package (Fox and Weisberg 2011; Bates et al. 2014), including the treatment as fixed effect.

Concerning the first reproductive event, we tested the probability of producing the first clutch with one generalised linear mixed effects model with binary logistic regression (i.e., 1-0; Harrell 2015). As the body weight of females is known to influence their fecundity (Sutton et al. 1983; Antoł and Czarnoleski 2018; Durand et al. 2018), we included the weight, the treatment, and their interaction as fixed effects. The time to produce the first clutch (i.e., number of days from the first contact with male to the delivery of offspring) and the number of offspring of the first clutch were modelled in two linear mixed effects models including the weight, the treatment, and their interaction as fixed effects. For the second reproductive event, we used three models like those concerning the first reproductive event. Since the first reproduction can influence the second reproduction by energy investment, we added the number of produced offspring in the first clutch as fixed effect, with the treatment, the weight and the interaction between the treatment and the weight.

The total number of offspring (first and second clutches included) was analysed with one linear mixed effects model including the treatment and the weight as fixed effects.

The haemocyte concentrations (number of cells per µL of haemolymph), the size of viable haemocytes and β-galactosidase activity were analysed using linear models with Gaussian distribution, and viability (proportion of viable haemocytes) using one generalised model with Binomial distribution (Harrison et al. 2018). Since the haemocyte parameters and senescence biomarkers were analysed both in females having one or two clutch(es), we used four mixed effects models, including the treatment, the number of clutches and the total number of offspring (first and second clutches included) as fixed effects. To deepen the interdependence of treatments and reproduction, we only allowed interactions of the treatment with the total number of offspring, and the treatments with the number of clutches.

For all models (i.e., survival, weight, probability of producing the first and second clutches, time to produce these clutches, the number of offspring in each clutch, haemocyte parameters and β-galactosidase activity), we entered the experimental replicates as random factor. This factor allows to correct the non-independence of samples within the same replicate of treatment (Harrison et al. 2018). Whether the treatment influenced the considered variable, we compared the pairs of means between each treatment by using Tukey adjustment (lsmeans packages; Lenth 2016). When interactions with treatment and weight were significant in our models, we performed the Pearson’s correlation test for each treatment, to obtain the effect of weight in each treatment.

The R script and the datasets used to perform our analysis are available on the open access repository Mendeley Data https://data.mendeley.com/datasets/gd24nvncvf/2

## ﻿Results

### ﻿Survival abilities

**Figure 2.**
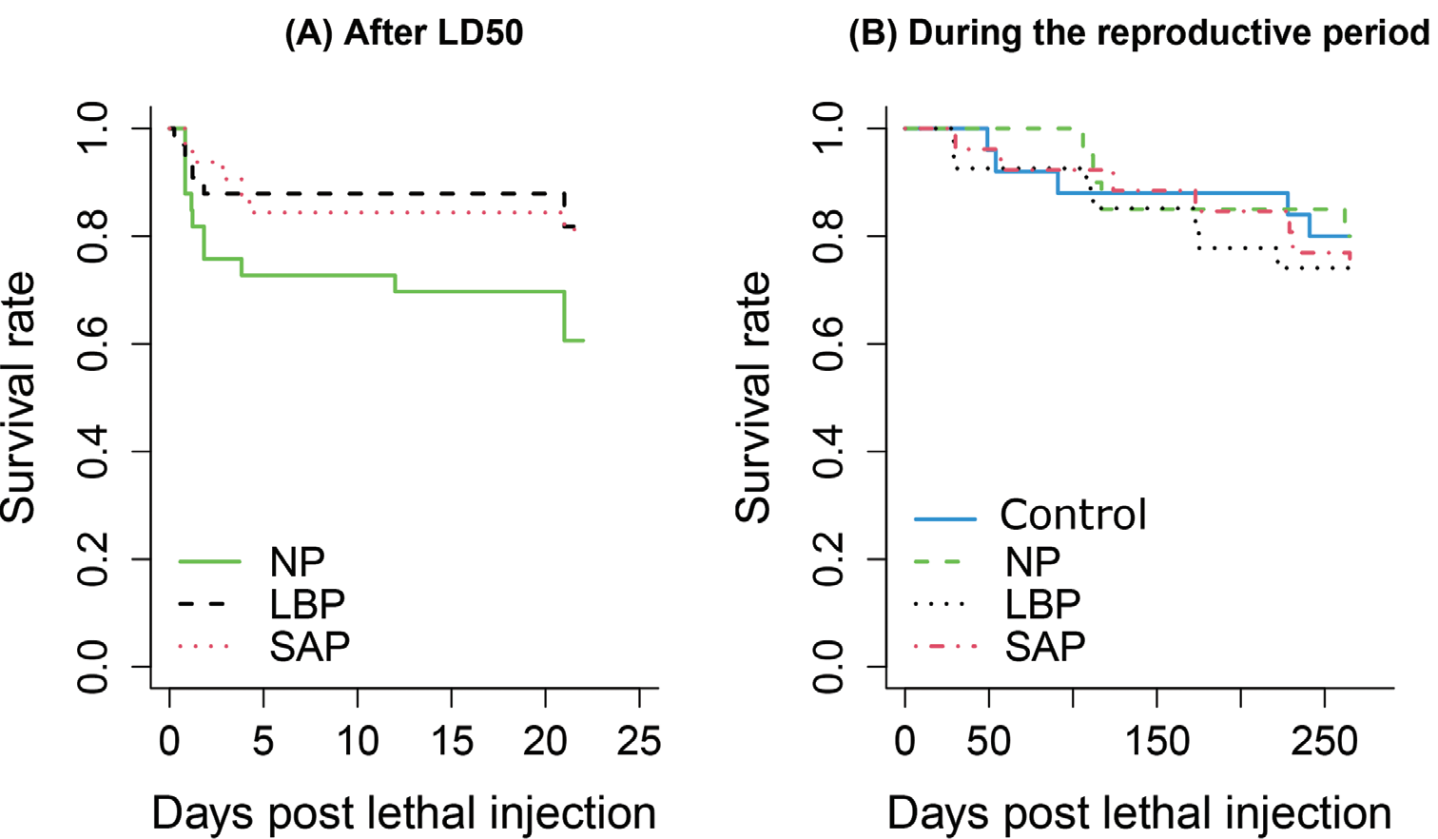
Survival rates **A** 22 days after the LD50 injection, and **B** during the reproductive period (ca. eight months). Abbreviations: NP: females non-primed in the priming procedure. LBP: females primed with sterile LB broth. SAP: females primed with 103 living *S.enterica*. Control: females that have never been injected. NP, LBP and SAP received the LD50 injection. Statistical results of comparisons between treatments are presented in Table S1.

**Figure 3.**
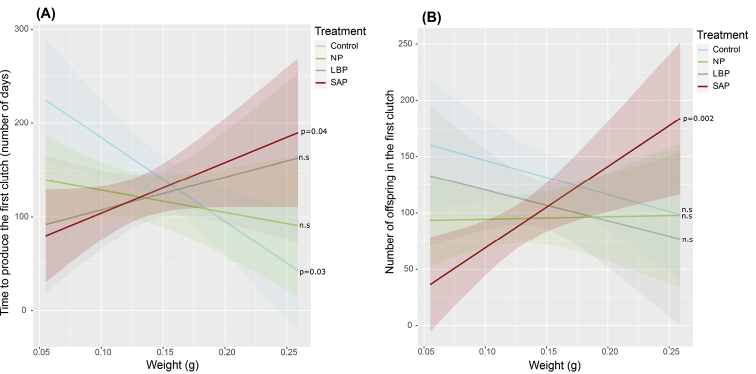
Interactions effects of body weight and treatment on **A** the time to produce the first clutch, and **B** the mean number of offspring in the first clutch per female. Abbreviations: control: never-injected females; NP: non-primed females; LBP: females primed with sterile LB broth, SAP: females primed with 103 living *S.enterica*. SAP, NP and LBP received the LD50 injection. P-values indicate a significant relationship between x and y axis of the considered treatment (Pearson’s correlation test).

The treatment had a slight effect on the survival abilities of females after the LD_50_ injection and before reproduction (*X*^2^ = 5.17, df = 2, p = 0.07; Fig. 2A). SAP and LBP females tended to better survive than NP females (p = 0.06 for each, Suppl. material 1: Table S1), with significant 59% lower risk of death for each (LBP: HR = 0.41, 95% CI = [0.15-1.1]; SAP: HR = 0.41, 95% CI = [0.16-1.1]). No difference in survival rates was observed between SAP and LBP females (Fig. 2A, Suppl. material 1: Table S1). During the reproductive period, the survival rates of females were not influenced by their treatment (*X*^2^ = 0.53, df = 3, p = 0.91; Fig. 3). Whatever the number of infections with *S.enterica*, injected females had same survival abilities with control females that have never been injected (Fig. 2B, Suppl. material 1: Table S1).

### ﻿Body weight of females

**Figure 4.**
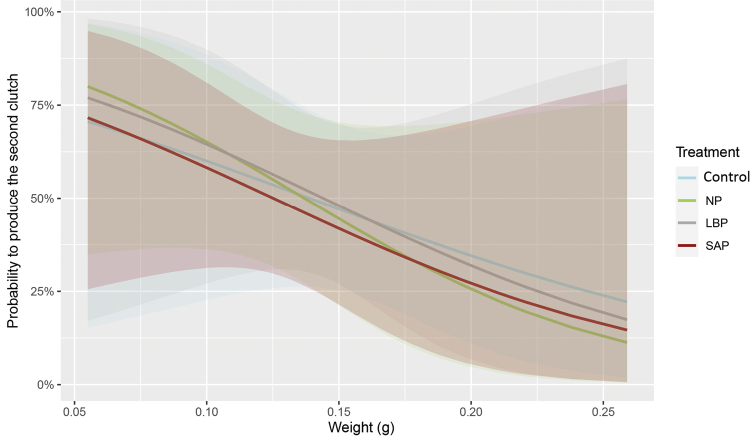
Probability to produce the second clutch according to female’s weight and treatment. Curves were calculated using average marginal effects of the absence/presence of the second clutch (0/1) related to the weight of females. Coloured distributions represent the confident interval for each treatment (95%). Abbreviations: control: never-injected females; NP: non-primed females LBP: females primed with sterile LB broth, SAP: females primed with 103 living *S.enterica*. SAP, NP and LBP received the LD50 injection.

The weight of females after the second infection with *S.enterica* (before reproduction) was influenced by their treatment (*X*^2^ = 8.05, df = 3, p = 0.04; Suppl. material 1: Fig. S1, Table S2). Even though the pairs of comparisons between females of the different treatments didn’t show significant effects, control females had an average weight 22% higher than that of NP females (Mean ± SE: control = 0.15g ± 0.008, NP = 0.12g ± 0.01; estimate = 0.03, SE = 0.01, df = 82.8, p = 0.09; Fig. 4) and SAP females (Mean ± SE: control = 0.15g ± 0.008, SAP = 0.12g ± 0.007, estimate = 0.02, SE = 0.01, df = 82.7, p = 0.07, Suppl. material 1: Fig. S1). No difference of weight was observed between the females of the other treatments (control vs. LBP: estimate = 0.01, SE = 0.01, df = 82.7, p = 0.46; NP vs. LBP: estimate = -0.01, SE = 0.01, df = 80.7, p = 0.76; control vs. SAP: estimate = -0.0007, SE = 0.0122, df = 81.0, p = 0.99; LB vs. SAP: estimate = 0.01, SE = 0.01, df = 81.5, p = 0.76).

## ﻿Reproduction

### ﻿First reproductive event

During the reproductive period, almost all females produced one clutch (SAP: 23/24, LBP: 20/22, NP: 22/22, control: 21/22). The probability to produce the first clutch was neither influenced by the treatment (*X*^2^ = 1.09, df = 3, p = 0.77) nor by the weight of females before reproduction (*X*^2^ = 0.18, df = 1, p = 0.66) or the interaction between the treatment and the weight (*X*^2^ = 2.61, df = 3, p = 0.45). Females were able to produce the first clutch regardless of their treatment or their weight before reproduction.

The time to produce the first clutch was neither influenced by the treatment (*X*^2^ = 1.15, df = 3, p = 0.76, Fig. 3) nor by the weight of females before reproduction (*X*^2^ = 0.49, df = 1, p = 0.48, Fig. 3). However, the interaction between treatment and weight showed a significant effect (*X*^2^ = 13.32, df = 3, p = 0.003, Fig. 3A). The time to produce the first clutch depended on the weight of females differently according to their treatment. In control females, lighter females took longer time to produce the first clutch (200 days on average) comparing to heavier control females (50 days on average; Pearson’s correlation: t = -2.34, df = 19, p = 0.03; Fig. 3A). Conversely, in SAP females, lighter females took less time to produce the first clutch (100 days on average) comparing to heavier SAP females (200 days on average; Pearson’s correlation: t = 2.13, df = 21, p = 0.04; Fig. 3A). In LBP and NP females, no difference in the time to produce the first clutch was observed according to their weight (LB: t = 1.17, df = 18, p = 0.25; NP: t = -0.82, df = 17, p = 0.42; Fig. 3A).

The number of offspring in the first clutch was not influenced by the female’s treatment (*X*^2^ = 7.07, df = 3, p = 0.06; Suppl. material 1: Fig. S2, Table S3), although control females produced an average of 134 offspring compared to an average of 90 offspring for NP and 95 offspring for SAP females (see Suppl. material 1: Fig. S2, Table S3 for Tukey’s comparisons). No significant effect was observed by the body weight (*X*^2^ = 0.40, df = 1, p = 0.52). However, the interaction between the body weight and the treatment influenced the number of offspring in the first clutch (*X*^2^ = 9.92, df = 3, p = 0.01; Fig. 3B). Hence, the number of offspring in the first clutch depended on the female’s weight differently for each treatment (Fig. 3B). Lighter SAP females produced less offspring than heavier SAP females (Pearson’s correlation test: t = 3.50, df = 21, p = 0.002; Fig. 3B), but the weight did not influence the number of offspring in the females of the other treatments (control: t = -1.12, df = 17, p = 0.27; NP: t = 0.09, df = 17, p = 0.92; LBP: t = -0.73, df = 18, p = 0.47; Fig. 3B).

### ﻿Second reproductive event

Among the females which produced the first clutch, half produced a second clutch, regardless of the treatment (control: 10/21; NP: 10/19; LBP: 10/20; SAP: 11/23). Hence, the probability to produce the second clutch was not influenced by their treatment (*X*^2^ = 0.19, df = 3, p = 0.97). This was neither influenced by the number of offspring in the first clutch (*X*^2^ = 0.20, df = 1, p = 0.64), nor by the interaction between the treatment and the weight (*X*^2^ = 0.14, df = 3, p = 0.98). However, the weight of the females influenced the probability of producing the second clutch (*X*^2^ = 4.60, df = 1, p = 0.03; Fig. 4), independently of the treatment. Lighter females had higher probability (74% on average for a weight of 0.06 g) to produce a second clutch comparing to heavier ones (30% on average for a weight of 0.20 g; Fig. 4; see Suppl. material 1: Table S4 for details).

The time to produce the second clutch (after the first one) and the number of offspring in the second clutch were influenced neither by the treatment (Time: *X*^2^ = 3.80, df = 3, p = 0.28; Number of offspring: *X*^2^ = 5.38, df = 3, p = 0.14), nor by the weight of females (Time: *X*^2^ = 0.97, df = 1, p = 0.32; Number of offspring: *X*^2^ = 0.54, df = 1, p = 0.45), the number of offspring in the first clutch (Time: *X*^2^ = 1.68, df = 1, p = 0.19; Number of offspring: *X*^2^ = 0.27, df = 1, p = 0.60), or the interaction between the treatment and the weight (*X*^2^ = 2.53, df = 3, p = 0.46). Regardless of their treatment, body weight, and cost of producing offspring in the first clutch, the females took the same time to produce the second clutch and produced a similar number of offspring in the second clutch.

### ﻿Total number of offspring

The total number of offspring (first and second clutch included) was not influenced by the treatment (*X*^2^ = 7.46, df = 3, p = 0.058). Even though control females produced an average of 190 offspring per female, compared to 131 offspring for SAP females (see Suppl. material 1: Fig. S3), no significant difference was observed by comparing the pairs of treatments (Suppl. material 1: Fig. S3, Table S5). The total number of offspring was neither influenced by body weight (*X*^2^ = 0.95, df = 1, p = 0.32), nor by the interaction between the treatment and the body weight of females (*X*^2^ = 0.95, df = 1, p = 0.31).

### ﻿Haemocyte parameters and senescence biomarkers

For the haemocyte concentrations, no significant effect of any fixed factors was observed (p > 0.05, see Suppl. material 1: Table S6 for details). Same results were obtained for the haemocyte viabilities (Suppl. material 1: Table S6). The number of infection(s) with *S.enterica* and the following reproductive event(s) did not impact the concentration or the viability of haemocytes.

**Figure 5.**
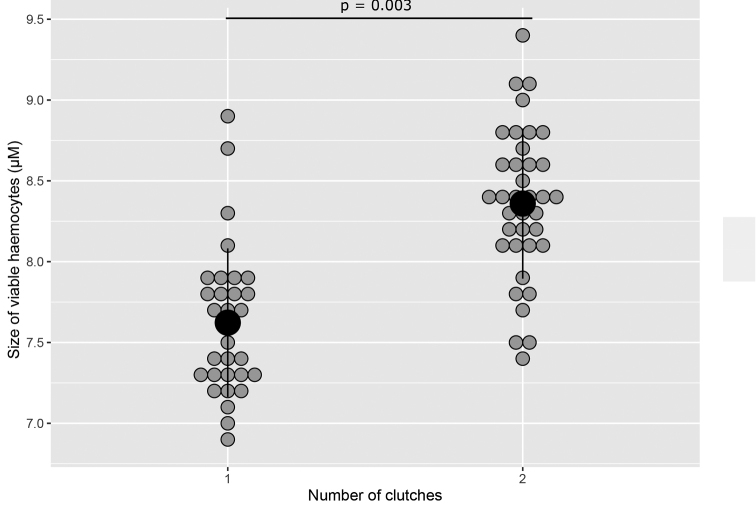
Size of viable haemocytes (µM) according to the number of clutch that females produced (1 or 2 clutches), all treatments combined.

Concerning the senescence biomarkers, the size of viable haemocytes was only influenced by the number of clutches that females produced, with an increase of the cell size in the case of a second clutch production (*X*^2^ = 12.99, df = 1, p = 0.003, Fig. 5). There was no influence of the other fixed factors (p > 0.05, see Suppl. material 1: Table S6). The β-galactosidase activity was not influenced by any of the fixed factors (p > 0.05, see Suppl. material 1: Table S6 for details). Whatever the treatment and the following reproductive event(s) of females, the β-galactosidase activities of females were similar.

## ﻿Discussion

**Figure 6.**
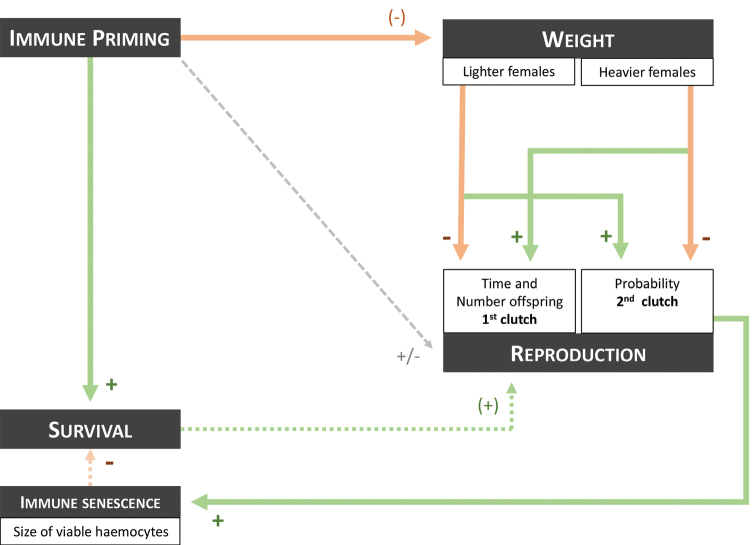
Summary diagram of the results. Orange arrows indicate a negative effect. Green arrows indicate a positive effect. If the corresponding sign is in brackets, the effect is a trend (0.05 < p < 0.10). +/- indicates no general effect.

Our study aimed to investigate the impact of immune priming with *S.enterica* (i.e., two consecutive infections with living pathogens) on the reproductive ability and senescence biomarkers of females of *A.vulgare*. Fig. 6 illustrates our main results.

### ﻿Immune priming improves the survival abilities but negatively impacts the mean body weight of females

As expected, we showed a protective effect of immune priming on female survival rates: the first encounter with *S.enterica* improves survival ability of females after the second and lethal infection, confirming previous results described in Prigot-Maurice et al. (2019, 2021). In surviving females, we showed a negative effect of immune priming on body weight of females (Fig. 6). After the LD_50_, females that received one or two infection(s) with *S.enterica* (NP or SAP) tended to be lighter than females that were never injected (control). Because *S.enterica* infection(s) is expected to induce metabolic costs related to immune functions like other pathogenic infections in invertebrates (Schwenke et al. 2016), the lower body weight of females could be explained by two non-exclusive hypotheses. It could either indicate (1) a decrease of the energetic resources of females (e.g., fat body), or (2) a slowdown in growth at the expense of investment in immunity, since body weight and size are closely related in *A.vulgare* (Antoł and Czarnoleski 2018; Durand et al. 2018; Schwenke et al. 2019). Interestingly, the injection of sterile LB Broth did not impact body weight of females, while these females have also been exposed to the second infection with living *S.enterica*. This result does not support the findings of other studies showing that individuals injected with non-pathogenic immunostimulants (e.g., LPS) became lighter as well (*T.molitor*, Moret 2006; *Hemedeinacrassidens* (Blanchard, 1851), Kelly 2011). In *A.vulgare*, the first immune stimulation without pathogens (LBP treatment) appears to prevent the energetic cost of the second infection with *S.enterica*. Similar results have been observed in *Cambarusclarkia* (Girard, 1852), for which individuals previously injected with live attenuated *Salmonella* do not show variation in body weight in the two months following infection (Ning et al. 2009).

### ﻿No evidence of immune priming costs on the reproductive abilities of double infected females

Since energetic investment in immunity often reduces available energy to produce offspring, the negative impact of immune responses on reproductive ability is widely observed across invertebrate species (Schwenke et al. 2016). While we hypothesised that the persistent infection of *S.enterica* during immune priming in *A.vulgare* would also negatively impact its reproductive abilities, we didn’t find any statistical evidence of lower probability to produce clutch(es) or lower number of offspring in females experiencing two consecutive infections with living *S.enterica* (SAP). All females infected (NP, LBP, and SAP) were as likely to reproduce as females that were never infected (control). In addition, the females that mounted immune priming (SAP) have similar survival rates during the reproductive period to females that have received only one (NP, LBP) or no injection (control females; Fig. 2B). Hence, females of *A.vulgare* maintain high survival rates with no apparent reproductive cost, while cellular changes are sustained for at least seven days, and *S.enterica* persists in the haemolymph of the females for fifteen days after the infections (Prigot-Maurice et al. 2019).

Most studies that investigated the costs of mounting immune priming showed a negative impact on reproduction (Schwenke et al. 2019). However, from an evolutionary point of view, a biological process inducing higher costs than benefits would be counter-selected. This counter-selection would be particularly strong when the biological process reduces the reproductive ability of individuals because it also reduces the possibility to transmit this process to the next generation. From this statement, it appears that immune priming should be selected during evolution if it does not induce a high cost (Moret 2003). Very few studies have shown absence of cost when investigating the evolutionary outcome of immune priming or transgenerational immune priming (Tetreau et al. 2019). In females of *T.molitor*, for instance, infection with *A.globiformis* or *B.thuringiensis* does not impact the number of eggs but increases the hatching rate of these eggs (Dhinaut et al. 2018). In the study of Bordoni et al. (2018), immune primed queen ants of *Crematogasterscutellaris* (Olivier, 1792) produced as many worker offspring as control queens. In addition, these ant workers displayed higher survival rates compared to workers derived from non-primed queens (Bordoni et al. 2018). By demonstrating that immune priming is not always associated with reproductive costs, these studies give important clues about the adaptive nature of immune priming. In our study, the absence of cost on immediate reproduction in female of *A.vulgare* expressing immune priming agrees with this but raises the question of how this immune process could be exempted from reproductive costs.

If the energetic resources of individuals are limited and trade-offs are inevitable between reproduction and immune response to infection (Schwenke et al. 2016), natural selection should favour the most optimal immune responses facing these allocations (Rauw 2012). To explain the reduction of reproductive costs following infection, we supposed that immune priming relies on increased tolerance against *S.enterica* rather than increased resistance, which would require much more energy at the expense of other biological functions (Zuk and Stoehr 2002; Rauw 2012). However, as stated by Tetreau et al. (2019), “absence of evidence does not always mean evidence of absence”. Indeed, no evidence of reproductive cost induced by immune priming does not mean there is no cost at all. It could result from the lack of statistical power and/or from methodological bias, where the costs are associated with other life-history traits that were not measured during the experiment. One important trait to consider is the total lifespan of females (i.e., their abilities to reproduce in later reproductive season), but more importantly, the life-history traits of the offspring of immune primed females. Immune priming is often associated with costs in the following generations, such as longer developmental time, reduced fecundity or reduced immune functions of offspring (for review see Tetreau et al. 2019). In *Crassostreagigas* (Thunberg, 1793), offspring from immune primed mothers take longer to develop, which delays their sexual maturity compared to offspring from non-primed mothers (Robinson and Green 2020). In the present study, the total number of offspring of females is not altered by double infections of *S.enterica*, but this does not prove that the fitness of these offspring is not negatively impacted. Hence, to conclude about the adaptive nature of immune priming in *A.vulgare*, it is required to look at the reproductive, growth and survival abilities against pathogens of offspring derived from females that have established immune priming during their lifetime. If the trans-generational benefits of immune priming in offspring are higher than costs, then immune priming in this species should be adaptive in an evolutionary sense.

### ﻿Indirect effect of immune priming on the first clutch production through the body weight of females

Even though we observed no evident cost on reproduction in females receiving the double infection of *S.enterica*, the treatment of females indirectly influences their reproductive strategies through body weight. In never-injected (control) females, the lighter ones took a longer time (200 days on average) to produce the first clutch than heavier ones (50 days on average; Fig. 3A). We suggest that this difference in body weight in control females is the result of a strategy to optimise reproduction in non-stressful conditions. Producing clutch is an expensive event in *A.vulgare*, because the production of the marsupium requires considerable energy by the female (Surbida and Wright 2001; Antoł and Czarnoleski 2018). However, the size of the marsupium is proportional to the size of the female, making the largest females the ones that normally produce more offspring by clutch (Antoł and Czarnoleski 2018). Because larger females *A.vulgare* have a higher fecundity (Waller and Verdi 2016; Durand et al. 2018), it is in the interest of females to grow before the reproductive event (under non-stressful conditions), also supported by the indeterminate growth ability of terrestrial isopods (Antoł and Czarnoleski 2018). By investing energy in their growth, thus delaying the production of the first clutch, lighter control females would indirectly invest in their reproduction by preparing the optimal physical conditions to produce as many offspring as possible in the following clutch (Lawlor 1976). We observed that lighter control females produced as many offspring in the first clutch as heavier control females (Fig. 3B), which supports the idea of an energetic investment in growth before reproduction. This result is in line with Warburg (2011), who demonstrated no relationship between the original body weight of females and the number of offspring in four different species of terrestrial isopods maintained in non-stressful conditions.

Otherwise, immune priming of double-infected females also induces different effects on the production of the first clutch according to body weight (Fig. 6). Indeed, two different strategies of reproduction were observed in females that experienced two consecutive infections with *S.enterica* (Figs 3, 6). The lighter SAP females took less time to produce the first clutch (100 days on average) but produced less offspring in this first clutch (50 offspring on average; Fig. 3) compared to heavier SAP females. In contrast, the heavier SAP females took more time to produce the first clutch (200 days on average), but it contained more offspring (150 offspring on average; Fig. 3).

From our point of view, these reproductive patterns in SAP females result from an alteration in energetic resource allocation. During a stressful event occurring in the lifetime of an organism, like an infection, it could opt for the investment of its remaining energy in reproduction, at the expense of growth, in order to maximise fitness before dying (Creighton et al. 2009). Hence, when females of *A.vulgare* have used a lot of energy in immunity against the double infection with *S.enterica* (i.e., are lighter), they probably have less remaining energy to invest in growth and/or to produce numerous offspring. The lower number of offspring in the first clutch of the lighter SAP females could thus be the consequence of constraints by marsupium size (i.e., it might also be smaller) and/or the reduction of available energy for numerous offspring (if marsupium size is similar with that of heavier females). For both possible cases, the lighter SAP females produced a lower number of offspring in the first clutch compared to the heavier ones, which probably requires less incubation time in the marsupium, thus reducing the number of days to produce the first clutch (Antol and Czarnoleski 2018). For the heavier SAP females, we assume that the costs of immunity during the infections of *S.enterica* were lower than those of lighter SAP females. As a result, heavier SAP females may have more energy for growth and/or to produce numerous offspring that take more time to complete their embryogenesis (Antol and Czarnoleski 2018). To test this hypothesis, it would be possible to supplement the food of SAP females with metabolites. For instance, aquatic crustaceans fed with Lysine, Arginine, or Threonine-rich diet display higher body weight and reproductive ability (for review: Huang et al. 2020). If SAP females of *A.vulgare* fed with supplemented diet gain in body weight and increase their reproductive abilities compared to poor-diet SAP females, then the lower reproductive ability of poor-diet SAP females will demonstrate the energetic allocation toward immunity to the detriment of reproduction (and maybe growth) in these lighter females.

These two different strategies illustrate a plasticity in resource allocation following two infections with *S.enterica* that depends on the investment of each female in the different physiological functions, namely somatic maintenance (including response to pathogens and/or growth) and reproduction. However, the total number of offspring per female (first and second clutches included) was influenced neither by the number of infections nor by body weight or the interaction between these parameters. Hence, whatever the allocation strategy of energetic resources in the first clutch in SAP females, the lighter of them mobilise enough energy to finally produce as many offspring as the heavier SAP females. This lack of effect seems explained by the second reproductive event.

### ﻿Heavier females have reduced probability to produce a second clutch, regardless of the number of infections

Concerning the second reproductive event, the probability of producing the second clutch only depended on the body weight of the females: the heavier the females are, the less likely they are to produce a second clutch (ca. 25%) compared to lighter females (ca. 75%). We suppose that the investment in the first clutch by heavier females is more expensive than for those lighter ones, regardless of treatment. As we have already stated, the costs of producing one marsupium are high, and positively correlated to female size (Dangerfield and Telford 1995; Lardies et al. 2004; Antoł and Czarnoleski 2018). For instance, Antol and Czarnoleski (2018) demonstrated that clutch size and clutch mass increased with female body mass in the terrestrial isopod *Porcellioscaber* (Latreille, 1804). In *Porcelliolaevis* (Latreille, 1804), the heavier females have higher metabolic rates during eggs incubation comparing to lighter ones (Lardies et al. 2004). Hence, we suppose that the heavier females may not have enough remaining energy (after the first clutch) to produce a second marsupium in the time course of our experiment. Here, we decided to stop the experiment at the end of the first reproductive season (ca. eight months after infections). The probability of producing a second clutch must be considered for this period. It does not mean that heavier females will not be able to produce other clutches in the second reproductive season. Several studies on crustaceans demonstrate that basal metabolic rates (estimated by protein synthesis and oxygen uptake) increase with individual body weight (Houlihan et al. 1990; Whiteley et al. 1996, 2001), indicating the allocation of energetic resources in somatic maintenance (Whiteley and Faulkner 2005). To confirm our previous hypothesis, the oxygen consumption and the rate of proteins synthesis could be used to compare the energetic costs of producing the first clutch between the heavier and the lighter females of *A.vulgare*.

Nevertheless, this result raises questions about the intrinsic factors that cause the production of one or two clutch(es) in females of *A.vulgare* depending on body weight, regardless of treatment. The first assumption about the lower probability to produce two clutches by heavier females is related to the production of better-quality offspring. According to theoretical predictions, females producing a single clutch should provide higher rates of care to their offspring than those producing several clutches (Tallamy and Brown 1999; Gilbert and Manica 2010, Meunier et al. 2012). In *Forficulaauricularia* (Linnaeus, 1758) for example, females providing more pronounced maternal care to the offspring in the first clutch are less likely to reproduce later (Koch and Meunier 2014). The quality of offspring is often measured through their size/weight at birth because it would improve survival abilities of offspring (Brody and Lawlor 1984; Dangerfield 1997), especially in species where the ability to grow is indeterminate, like in terrestrial isopods (Hubbel 1971; Lawlor 1976; Dangerfield and Telford 1995). In nine different species of isopods, there is evidence of positive correlation between female size and offspring size, thus potentially offspring quality (Antol and Czarnoleski 2018). In *A.vulgare*, females maintained in a resource-poor environment produce larger offspring compared to females maintained in usual environment (Brody and Lawlor 1984). However, a greater investment of parents in offspring size, does not always increase the fitness of their offspring (Depeux et al. 2020a). In *A.vulgare*, older parents produce larger and more numerous offspring than younger ones, but the offspring of these older parents have lower survival abilities and are unable to reproduce (Depeux et al. 2020a). Complementary experiments are thus needed to evaluate the quality of offspring of females producing only one clutch, by following their survival and reproduction in the adult stage (Depeux et al. 2020a).

The second assumption concerning the probability to produce one or two clutches refers to the environmental and physiological conditions of the females before reproduction. By observing the reproductive phenology in four different species of terrestrial isopods, Dangerfield and Telford (1995) postulated that: “the tactic of repeated reproduction, with a relatively conservative allocation of resources to each reproductive event to enhance survival probabilities, would be favoured, particularly in an environment where juvenile mortality and the chance of complete brood failure is high”. The likelihood of clutch failure and offspring mortality could be sensed by females when the environmental and physiological conditions vary, such as the increase/decrease of temperature, food intake (i.e., energetic resources), or pathogenic infections (Brody and Lawlor 1984; Dangerfield and Hassall 1992; Dangerfield and Telford 1995; Hassall et al. 2005). In the case of pathogenic infections in invertebrates, for instance, level and frequency of pathogens and danger-associated molecular patterns may indicate the pathogen’s abundance and persistence in the environment where the offspring will be produced. Hence, the lower weight of females, maintained in infectious conditions or not, could also be a signal to promote individual fitness, as in many species (Mousseau 1998; Kim et al. 2017). If the lighter females have less available energy for their offspring for a given time or have smaller marsupia that constraint the oviposition of numerous eggs, then they may counterbalance the lower number of offspring in the first clutch by producing a second clutch as soon as they acquire enough energy to do so. Our results about the lighter females experiencing two consecutive infections with living *S.enterica* (i.e., that sense environmental and physiological stressful conditions) confirm this assumption. With their higher probability to produce a second clutch compared to heavier females, they produce a similar total number of offspring as the heavier females that, however, produce only one clutch.

### ﻿While immune priming neither impacts the haemocyte parameters nor the senescence patterns after reproduction, producing two clutches increases the cellular senescence of females

For the senescence biomarkers, we only observed an effect of the investment in reproduction on the size of the viable haemocytes: regardless of treatment, females that have produced a second clutch had larger viable haemocytes than those of females that produced only one clutch. The size of haemocytes in *A.vulgare* increases with the age of individuals, making this morphometric trait a biomarker of senescence (Depeux et al. 2020b). Here, for an identical age of females, the larger size of haemocytes could therefore show premature senescence in females that produced two clutches, suggesting a decrease in total lifetime. This result could illustrate a trade-off between the energy allocated to reproduction or somatic maintenance. To confirm this cellular senescence state, other biomarkers of senescence should be analysed, as the TERT gene expression (Depeux et al. 2020b), or the telomere length for instance. However, the higher viable cell size could also indicate different proportion of haemocyte types. In *A.vulgare*, hyalin and semi-granular haemocytes are taller than granular haemocytes (Chevalier et al. 2011). Hence, females producing two clutches could have higher proportion of hyalin and semi-granular haemocyte types comparing to females producing only one clutch, but further experiments (with cytometric analysis for instance), are also needed to confirm this hypothesis.

Finally, we observed no effect of the treatment or the reproductive event(s) on the haemocyte parameters of females (concentration and viability). We conclude that several months after *S.enterica* infection(s), immune cell production is no longer impacted by infection(s) (Prigot-Maurice et al. 2019), nor by the following reproductive event(s). However, these immune parameters only reflect the quantity and basal viability of haemocytes of the females. This does not mean that the immune activities of these haemocytes (i.e., the immunocompetence of females against a future threat) is not impacted by infectious and reproductive events (Lawniczak et al. 2007), since the females that have produced two clutches displayed higher cellular senescence pattern through the size of their viable haemocytes.

## ﻿Conclusions

Our study aimed to investigate the impact of immune priming with *S.enterica* on life-history traits and senescence biomarkers in *A.vulgare*. While current studies in various species show negative effects of immune priming, we only found an indirect effect of immune priming by body weight of females that could indirectly impact reproduction. However, we observed no strong effects of consecutive infections with *S.enterica* in the reproductive ability of female. Even though the absence of evidence for cost(s) does not mean that there is no cost at all, the fact that only a few studies reporting the absence of costs of immune priming or transgenerational immune priming could be explained by the difficulty to publish non-significant results. The publishing bias towards significant results of immune priming costs can change our view of evolutionary implications (Tetreau et al. 2019). Hence, our study tends to highlight the adaptive potential of immune priming in *A.vulgare* during evolution. Because *A.vulgare* is a detritivore and gregarious organism living for several years in moist environments particularly rich in microbial density and diversity (Warburg et al. 1984; Ranjard and Richaume 2001; Zimmer 2002; Broly et al. 2013; Bredon et al. 2020), the decreasing cost of immune priming on reproduction in an evolutionary scale for this species suggests its important role. Overall, our results tend to show that immune priming is not always associated with significant reproductive costs, even if the underlying mechanism is a sustained immune response lasting from the first to the second infection (Prigot-Maurice et al. 2019). Increasing studies on this subject will shed light on evolutionary mechanisms that have favoured immune priming over time.

## Supplementary materials

Supplementary material 1 Tables S1–S4, Figures S1–S3 Data type Pdf file. Explanation note **Table S1**. Pairwise comparisons (Tukey adjustment) of survival rates according to females’ treatments (NP: non-primed females; LBP: females primed with sterile LB broth; SAP: females primed with 103 S.enterica in the priming procedure; Control: never-injected females). Figure S1. Body weight of females before reproduction according to their priming treatment. NI: never-injected, control females; NP: non-primed females; LBP: females primed with sterile LB broth, SAP: females primed with 103 living S.enterica. NP, LBP and SAP females received the LD50 injection. Mean ± SE: Control = 0.15g ± 0.008, NP = 0.12g ± 0.01, LBP = 0.14g ± 0.007, SAP = 0.12g ± 0.007). Table S2. Pairwise comparisons (Tukey adjustment) of body weight of females before reproduction, according to the treatments (NP: non-primed females; LBP: females primed with sterile LB Broth; SAP: females primed with 103 S.enterica during the priming procedure; Control: never-injected females). **Figure S2**. Number of offspring in the first clutch according to the females’ treatment. NI: never-injected, control females; NP: non-primed females; LBP: females primed with sterile LB broth, SAP: females primed with 103 living S.enterica. NP, LBP and SAP females received the LD50 injection. **Table S3**. Pairwise comparisons (Tukey adjustment) of the number of offspring in the first clutch according to females’ treatments (NP: non-primed females; LBP: females primed with sterile LB Broth; SAP: females primed with 103 S.enterica during the priming procedure; Control: never-injected females). **Figure S3**. Total number of offspring (first and second clutches included) according to the females’ treatment. NI: never-injected, control females; NP: non-primed females; LBP: females primed with sterile LB broth, SAP: females primed with 103 living S.enterica. NP, LBP and SAP females received the LD50 injection. **Table S4**. Average marginal effects of the interaction between the probability to produce a second clutch and the body weight of females by treatment (NP: non-primed, Control females; LBP: females primed with sterile LB Broth; SAP: females primed with 103 S.enterica in the priming procedure; Control: never-injected females). **Table S5**. Pairwise comparisons (Tukey adjustment) of the total number of offspring according to females’ treatments (NP: non-primed, Control females; LBP: females primed with sterile LB Broth; SAP: females primed with 103 S.enterica in the priming procedure; Control: never-injected females). **Table S6**. Statistical results of haemocyte parameters and senescence biomarkers analysis (generalized linear mixed effect models). https://binary.pensoft.net/file/685586This dataset is made available under the Open Database License (http://opendatacommons.org/licenses/odbl/1.0/). The Open Database License (ODbL) is a license agreement intended to allow users to freely share, modify, and use this Dataset while maintaining this same freedom for others, provided that the original source and author(s) are credited.Cybèle Prigot-Maurice, Charlotte Depeux, Hélène Paulhac, Christine Braquart-Varnier, Sophie Beltran-Bech
