# Supplementary material for: ﻿Immune priming in Armadillidiumvulgare against Salmonellaenterica: direct or indirect costs on life history traits?
Source: Zookeys. 2022 May 18;1101:131–58. doi: 10.3897/zookeys.1101.77216 (PMC9848923; doi:10.3897/zookeys.1101.77216)
Supplement: Supplementary material 1 — Tables S1–S4, Figures S1–S3 [file zookeys-1101-131_article-77216__-s001.pdf]

Immune priming in *Armadillidium vulgare* against *Salmonella enterica*: direct or indirect costs on life history traits?

Cybèle Prigot-Maurice[1*], Charlotte Depeux[1*], Hélène Paulhac[1], Christine Braquart-Varnier[1#], Sophie Beltran-Bech[1#]

[1] : Université de Poitiers, Laboratoire Écologie et Biologie des Interactions, UMR CNRS 7267, 3 rue Jacques Fort, TSA 51106, F-86073 POITIERS Cedex 9, France.

* Co-first authors

Co-last authors

Corresponding author: Cybèle Prigot-Maurice (cybele.prigot@gmail.com)

Supplementary material

Figure S1. Body weight of females before reproduction according to their priming treatment. NI: never-injected, control females; NP: non-primed females; LBP: females primed with sterile LB broth, SAP: females primed with 10^3 living *S. enterica*. NP, LBP and SAP females received the LD_{50} injection. Mean ± SE: Control = 0.15g ± 0.008, NP = 0.12g ± 0.01, LBP = 0.14g ± 0.007, SAP = 0.12g ± 0.007).

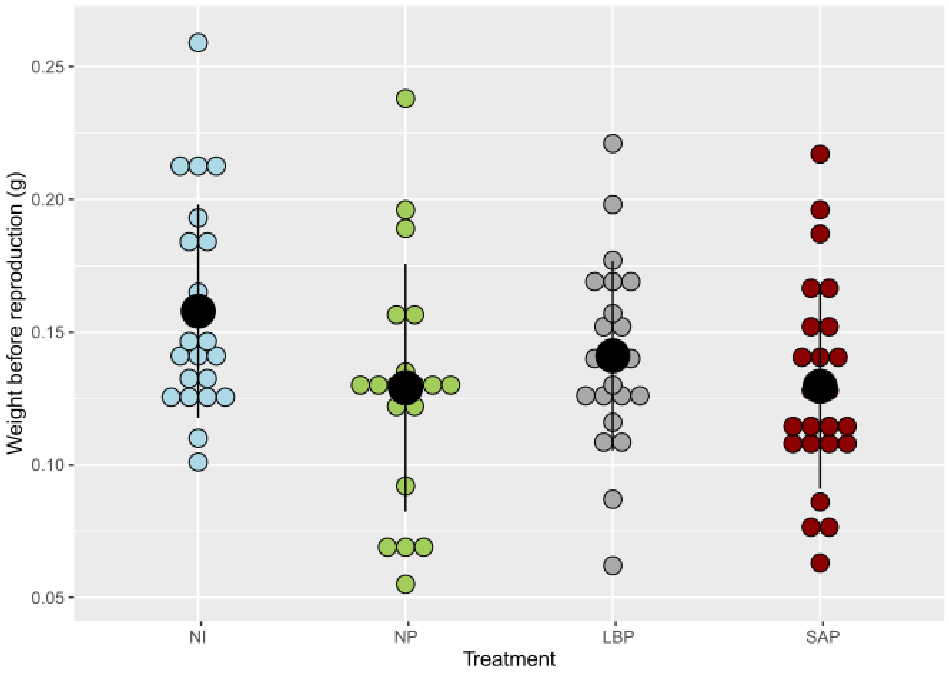

Figure S2. Number of offspring in the first clutch according to the females' treatment. NI: never-injected, control females; NP: non-primed females; LBP: females primed with sterile LB broth, SAP: females primed with 10^3 living *S. enterica*. NP, LBP and SAP females received the LD_{50} injection.

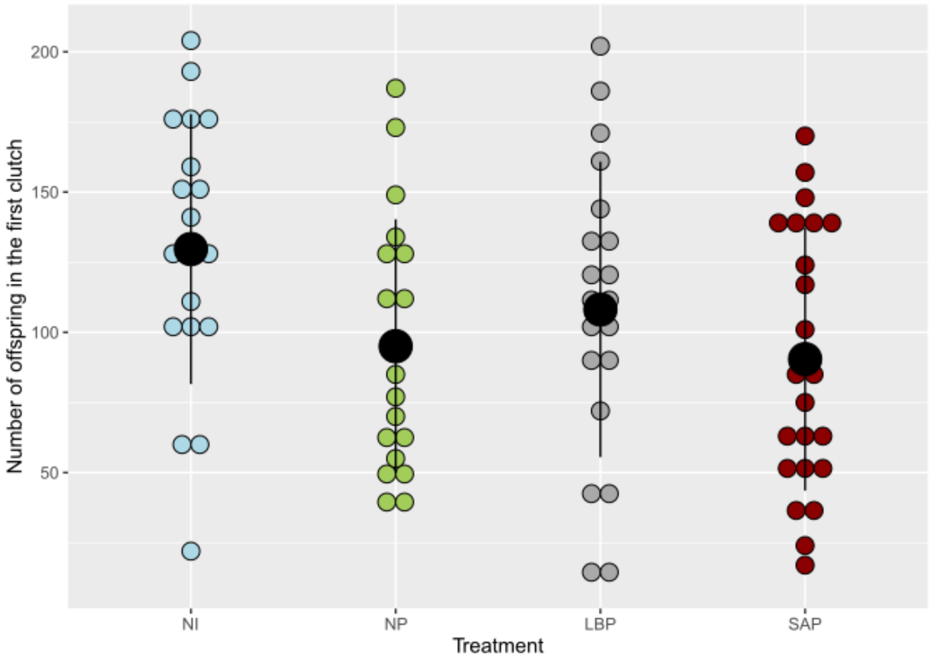

Figure S3. Total number of offspring (first and second clutches included) according to the females' treatment. NI: never-injected, control females; NP: non-primed females; LBP: females primed with sterile LB broth, SAP: females primed with 10^3 living *S. enterica*. NP, LBP and SAP females received the LD_{50} injection.

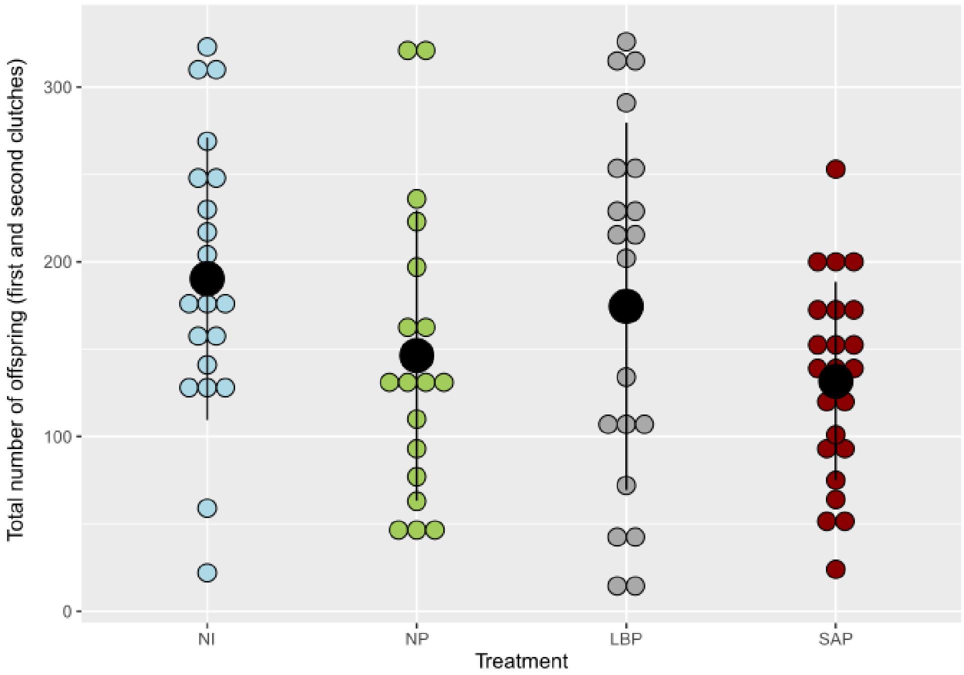

Table S1. Pairwise comparisons (Tukey adjustment) of survival rates according to females' treatments (NP: non-primed females; LBP: females primed with sterile LB broth; SAP: females primed with 10^3 *S. enterica* in the priming procedure; Control: never-injected females).

**SURVIVAL RATES (frequency of living females)**			
**22 days after LD_{50}**	estimate	SE	p-value
NP vs. LPB	0.914	0.494	**0.064**
NP vs. SAP	0.912	0.495	**0.065**
LBP vs. SAP	0.578	0.578	0.99
**During the reproductive period**	estimate	SE	p-value
NP vs. LPB	-0.33	0.627	0.952
NP vs. SAP	0.318	0.627	0.957
LBP vs. SAP	0.01	0.535	1.00
Control vs. NP	0.01	0.671	1.00
Control vs. LBP	-0.31	0.586	0.951
Control vs. SAP	-0.299	0.586	0.956

Table S2. Pairwise comparisons (Tukey adjustment) of body weight of females before reproduction, according to the treatments (NP: non-primed females; LBP: females primed with sterile LB Broth; SAP: females primed with 10^3 *S. enterica* during the priming procedure; Control: never-injected females).

**WEIGHT OF FEMALES**			
	estimate	SE	p-value
NP vs. LPB	0.01	0.012	0.76
NP vs. SAP	-0.0007	0.012	0.99
LBP vs. SAP	0.01	0.011	0.76
Control vs. NP	0.03	0.012	**0.09**
Control vs. LBP	0.01	0.012	0.46
Control vs. SAP	0.02	0.012	**0.07**

Table S3. Pairwise comparisons (Tukey adjustment) of the number of offspring in the first clutch according to females' treatments (NP: non-primed females; LBP: females primed with sterile LB Broth; SAP: females primed with 10^3 *S. enterica* during the priming procedure; Control: never-injected females).

NUMBER OF OFFSPRING IN THE FIRST CLUTCH			
	estimate	SE	p-value
NP vs. LPB	-14.37	15.4	0.78
NP vs. SAP	-2.21	14.8	0.99
LBP vs. SAP	12.16	14.8	0.84
Control vs. NP	39.38	16.4	**0.08**
Control vs. LBP	25.01	15.9	0.40
Control vs. SAP	37.18	15.7	**0.09**

Table S4. Average marginal effects of the interaction between the probability to produce a second clutch and the body weight of females by treatment (NP: non-primed, Control females; LBP: females primed with sterile LB Broth; SAP: females primed with 10^3 *S. enterica* in the priming procedure; Control: never-injected females).

MEAN WEIGHT = 0.06 g

Treatment	Predicted probability of second clutch	95% CI
Control	0.70	[0.16, 0.96]
NP	0.79	[0.35, 0.96]
LBP	0.76	[0.18, 0.98]
SAP	0.71	[0.26, 0.94]

MEAN WEIGHT = 0.20 g

Treatment	Predicted probability of second clutch	95% CI
Control	0.35	[0.11, 0.70]
NP	0.26	[0.05, 0.71]
LBP	0.32	[0.07, 0.76]
SAP	0.27	[0.06, 0.71]

Table S5. Pairwise comparisons (Tukey adjustment) of the total number of offspring according to females' treatments (NP: non-primed, Control females; LBP: females primed with sterile LB Broth; SAP: females primed with 10^3 *S. enterica* in the priming procedure; Control: never-injected females).

TOTAL NUMBER OF OFFSPRING (first and second clutches included)			
	estimate	SE	p-value
NP vs. LPB	-35.5	27.2	0.56
NP vs. SAP	5.5	26.2	0.99
LBP vs. SAP	41.0	26.1	0.40
Control vs. NP	56.7	28.7	0.20
Control vs. LBP	21.2	28.0	0.87
Control vs. SAP	62.2	27.4	**0.11**

Table S6. Statistical results of haemocyte parameters and senescence biomarkers analysis (generalized linear mixed effect models).

HAEMOCYTE CONCENTRATION			
	X^2	df	p-value
Treatment	2.77	3	0.42
Total number of offspring	0.00	1	0.99
Number of clutches	0.16	1	0.68
Treatment*Total number of offspring	1.56	3	0.66
Treatment*Number of clutches	0.52	3	0.91

HAEMOCYTE VIABILITY			
	X^2	df	p-value
Treatment	1.04	3	0.79
Total number of offspring	1.15	1	0.28
Number of clutches	0.11	1	0.73
Treatment*Total number of offspring	0.67	3	0.87
Treatment*Number of clutches	0.91	3	0.82

SIZE OF VIABLE HAEMOCYTES			
	X^2	df	p-value
Treatment	2.60	3	0.55
Total number of offspring	0.64	1	0.64
**Number of clutches**	**12.99**	**1**	**0.003**
Treatment*Total number of offspring	2.71	3	0.43
Treatment*Number of clutches	3.62	3	0.30

β-GALACTOSIDASE ACTIVITY			
	X^2	df	p-value
Treatment	7.10	3	0.06
Total number of offspring	0.46	1	0.49
Number of clutches	0.08	1	0.76
Treatment*Total number of offspring	2.42	3	0.48
Treatment*Number of clutches	2.24	3	0.52
